# A severe red tide (Tampa Bay, 2005) causes an anomalous decrease in biological sound

**DOI:** 10.1098/rsos.150337

**Published:** 2015-09-16

**Authors:** Katherine L. Indeck, Peter Simard, Shannon Gowans, Susan Lowerre-Barbieri, David A. Mann

**Affiliations:** 1 Eckerd College, 4200 54th Avenue South, St. Petersburg, FL 33711, USA; 2 College of Marine Science, University of South Florida, 140 7th Avenue South, St. Petersburg, FL 33701, USA; 3 Florida Fish and Wildlife Conservation Commission, 100 8th Avenue Southeast, St. Petersburg, FL 33701, USA; 4 Loggerhead Instruments, 6576 Palmer Park Circle, Sarasota, FL 34238, USA

**Keywords:** harmful algal bloom, *Karenia brevis*, ambient noise, snapping shrimp, fish chorusing, acoustics

## Abstract

Although harmful algal blooms (HABs) are known to cause morbidity and mortality in marine organisms, their sublethal effects are poorly understood. The purpose of this study was to compare ambient noise levels during a severe HAB event in Tampa Bay, Florida, to those during non-HAB periods. Passive acoustic monitoring was conducted using bottom-mounted autonomous acoustic recorders during a severe HAB in summer 2005, and in summers 2006, 2011 and 2012 (non-severe HAB years). Ambient noise levels were significantly higher during the non-HAB years due to an abundance of snapping shrimp (*Alpheidae*) sounds and fish chorusing. The difference of sound intensity between the study years is most likely attributable to effects of the HAB on the abundance and/or behaviour of fish and snapping shrimp as a result of mortality and stress-induced behavioural modifications.

## 1. Introduction

Harmful algal blooms (HABs or ‘red tides’) are characterized by the proliferation of toxic microscopic algae. In Florida (USA), HABs are common and frequently caused by the dinoflagellate *Karenia brevis,*which produces potent neurotoxins known as brevetoxins. Brevetoxins can affect the central nervous system of marine organisms by interfering with proper nerve transmission [1,2], causing morbidity and mortality [1,3]. In 2005, Tampa Bay, Florida, and its adjacent waters experienced a particularly severe HAB event, which was manifested by massive fish kills, benthic invertebrate die offs and unusual mortality of sea turtles and marine mammals [3–5].

Many species of marine animals produce sound. For example, over 800 species of fish are known to be soniferous (sound producing) [6], and snapping shrimp (*Alpheidae*) are recognized as dominant contributors to ambient noise levels in tropical and subtropical waters around the world [7]. Tampa Bay and its surrounding waters normally have considerable biological sound levels from fish chorusing and snapping shrimp [8,9]. The purpose of this study was to compare ambient noise levels between the severe 2005 HAB year and several non-severe HAB periods in an effort to assess whether there was a significant difference in biological sound production during the HAB.

## 2. Material and methods

Recordings were collected on the northern edge of Bunces Pass, a narrow channel connecting Boca Ciega (lower Tampa Bay) with the Gulf of Mexico. Autonomous acoustic recorders were bottom mounted at approximately 5 m depth from June to September of 2005 (severe HAB year), 2006, 2011 and 2012 (non-severe HAB years). All recordings for this study were collected at approximately the same location (27^°^39.14^′^ N, 82^°^44.65^′^ W), and each year several recorders were used in succession due to memory and battery constraints. In 2005 and 2006, recorders were Toshiba Pocket PCs with analogue to digital converters (22 kHz sample rate, 16 bit resolution) with a gain of 6 dB. Recordings had a duty cycle of 8 or 10 s every 10 min, and hydrophones were HTI-96-MIN, nominal sensitivity −164 dBV μPa^−1^. In 2011 and 2012, Loggerhead Instruments Digital SpectroGram recorders were used, operating with a 50 kHz sample rate and 16 bit resolution, with a gain of 20 dB. Recordings were at a duty cycle of 10 s per 10 min, and hydrophones were HTI-96-MIN, nominal sensitivity −170 dBV μPa^−1^.

All files were manually inspected (both visually and aurally) in Adobe Audition (2048 point spectrogram) to identify biological sounds. Using MATLAB, ambient noise levels (from two 4000 Hz wide bands: 50–4050 Hz and 5000–9000 Hz) were calculated as the monthly mean root mean square (RMS) amplitude. Only files recorded from 22.00 h to 04.00 h were analysed, to control for vessel noise (which is normally absent in Bunces Pass during this time) and to select for the period of optimum biological sound production [7,10]. Other sources of non-biological sounds (e.g. wind, rain) were considered equal across years and negligible for analysis. Using month, year and HAB presence (1/0) as independent variables, a multiple regression was run to predict RMS sound levels for each of the two bandwidths (the low frequency band encompassing fish sound production, the high frequency band representing snapping shrimp sound). The mean spectrum level (dB re 1 μPa^2^ Hz^−1^) was calculated for each month in MATLAB.

## 3. Results

**Figure 1.**
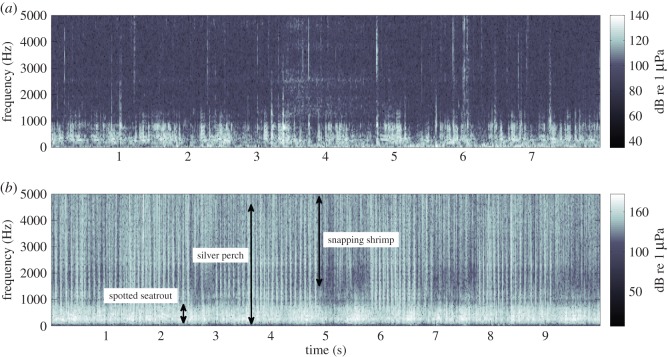
Spectrograms showing ambient noise for midnight August 15 (*a*) 2005 (severe HAB) and (*b*) 2006 (no severe HAB), 0–5000 Hz to show detail of fish sound. Approximate bandwidth of sounds from snapping shrimp, silver perch and spotted seatrout shown on 2006 spectrogram. Note some spotted seatrout and snapping shrimp present in 2005. Spectrograms are 512 point with a 50% overlap Hamming window.

**Figure 2.**
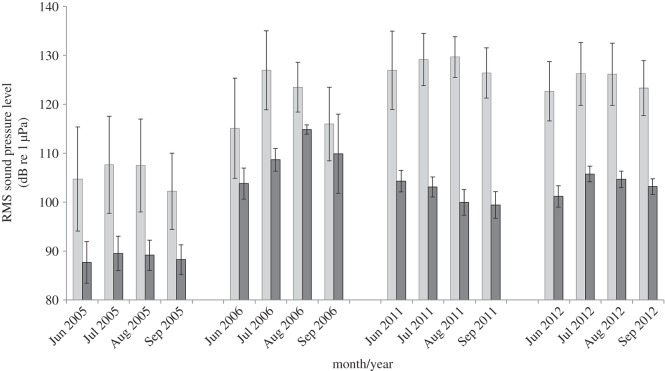
Bar graph showing RMS levels over the entire study period for the low frequency (50–4050 Hz) (light grey) and high frequency (5000–9000 Hz) (dark grey) sound bands.

**Figure 3.**
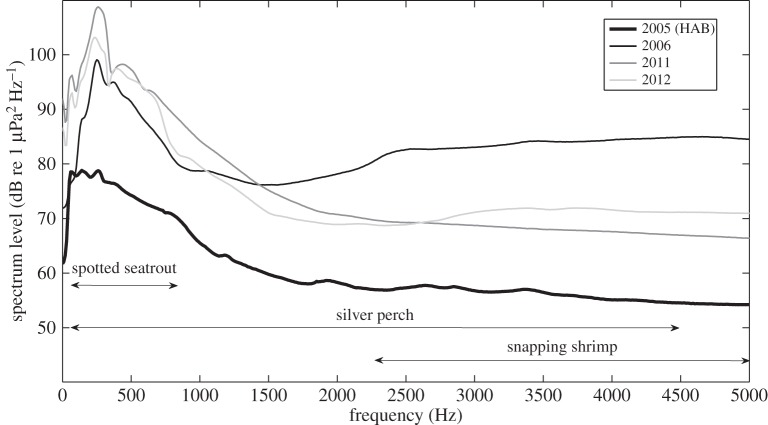
Energy spectra of mean August background noise for 2005, 2006, 2011 and 2012 (FFT resolution 10 Hz, 50 Hz high-pass filter), 0–5000 Hz to show detail of fish sound. Approximate bandwidth of sounds from snapping shrimp, silver perch and spotted seatrout indicated.

During manual inspection of spectrograms, the presence of snapping shrimp sound [7] and fish chorusing (especially by silver perch, *Bairdiella chrysoura*, and spotted seatrout, *Cynoscion nebulosus* [9,11]) was noticeably lower in 2005 (HAB year) than in 2006, 2011 and 2012 (non-HAB years) (figure 1). In 2005 (HAB year), monthly mean ambient noise levels ranged from 102.2 to 107.7 (low band) and from 87.7 to 89.5 (high band) dB re 1 μPa, whereas in non-HAB years (2006, 2011 and 2012) mean ambient noise levels ranged from 115.1 to 129.7 (low band) and from 99.4 to 114.8 (high band) dB re 1 μPa (table 1; figure 2). All three variables used for regression statistically significantly predicted RMS sound levels for each 4000 Hz band (low: *p*<0.001, *R*^2^=0.509; high: *p*<0.001, *R*^2^=0.684). HAB presence and year had the highest predictive values, while month had little predictive power (table 2). The energy spectra of the background noise from August 2005, 2006, 2011 and 2012 indicated noticeably depressed sound levels in 2005 in frequency bands of the sounds of snapping shrimp, silver perch and spotted seatrout (figure 3). In 2006, the year immediately following the severe HAB, sounds from snapping shrimp were particularly high in the acoustic recordings. However, fish sounds increased far more in 2011–2012, when snapping shrimp were not as loud (figures 2 and 3). This rebound and subsequent decline of snapping shrimp sound is probably responsible for the negative beta value for the high frequency band's year variable (table 2).

**Table 1.** Summary of monthly mean ambient sound levels.

		mean sound level (dB re 1 μPa) ± s.d.	
year	month	low (50–4050 Hz)	high (5000–9000 Hz)
2005	June	104.7±10.6	87.7±4.2
	July	107.7±9.9	89.5±3.5
	Aug	107.5±9.5	89.2±3.1
	Sep	102.2±7.8	88.3±3.0
2006	June	115.1±10.2	103.8±3.2
	July	126.9±8.1	108.7±2.3
	Aug	123.5±5.1	114.8±0.9
	Sep	116.0±7.5	109.9±8.1
2011	June	127.0±8.0	104.3±2.2
	July	129.1±5.3	103.1±2.0
	Aug	129.7±4.2	100.0±2.6
	Sep	126.4±5.1	99.4±2.7
2012	June	122.7±6.1	101.2±2.2
	July	126.2±6.4	105.7±1.6
	Aug	126.1±6.4	104.7±1.7
	Sep	123.3±5.6	103.2±1.6

**Table 2.** Regression summary for (*a*) low and (*b*) high sound bands, including both unstandardized (*B*) and standardized (*β*) regression coefficients.

	*β*	s.e. *β*	*B*	s.e. *B*	*t* (16 803)	*p*
(*a*)^a^						
intercept			−2767.40	52.20	−53.02	0.000
year	0.39	0.01	1.44	0.03	55.62	0.000
month	−0.06	0.01	−0.61	0.06	−10.64	0.000
HAB	−0.40	0.01	−11.22	0.19	−58.69	0.000
(*b*)^b^						
intercept			2528.62	32.41	78.02	0.000
year	−0.42	0.01	−1.21	0.02	−75.01	0.000
month	0.02	0.00	0.15	0.04	4.13	0.000
HAB	−1.00	0.01	−21.91	0.12	−184.65	0.000

^a^*R*=0.71. *R*^2^=0.51. Adjusted *R*^2^=0.51. *F*_3,16 803_=5809.5,*p*<0.001, s.e. of estimate =7.70.

^b^*R*=0.83. *R*^2^=0.68. Adjusted *R*^2^=0.68. *F*_3,16 803_=12 125.0,*p*<0.001, s.e. of estimate =4.78.

## 4. Discussion

This study quantitatively compared ambient noise levels during a severe HAB event in 2005 to those during non-HAB years. The study site was continuously exposed to high cell counts of *K. brevis* for the duration of the 2005 recordings (fig. 1 in [12]). The significantly decreased ambient noise in 2005 appears to be due to the near absence of fish chorusing (especially by silver perch and spotted seatrout) and snapping shrimp sound that was routinely observed in 2006, 2011 and 2012. The reduction in biological sound is most likely attributable to effects of the HAB on the abundance, distribution and/or behaviour of these animals.

Crustaceans have generally been considered less susceptible to brevetoxicosis than other marine invertebrate groups [13,14]. However, several studies have found that different species exhibit varying degrees of behavioural responses to brevetoxin exposure, ranging from low sensitivity to severe behavioural abnormalities [14–16]. In addition, a mass mortality event of benthic crustaceans (and other invertebrates) in Tampa Bay was associated with the 2005 HAB [3]. Although this mortality was attributed to anoxic conditions resulting from increased biological oxygen demands associated with the HAB, tissue samples contained very high concentrations of brevetoxins [3]. Therefore, although there have been no studies documenting the impacts of HABs on the behaviour or mortality of snapping shrimp, it is reasonable to suggest that the 2005 HAB event was responsible for the lack of snapping shrimp detected acoustically that summer.

Similarly, the observed decrease of fish chorusing was probably a result of HAB-related mortality and/or stress-induced behavioural modifications. In 2005, the severe HAB was responsible for an unusually high number of fish kills in Tampa Bay [4,12] and adjacent areas [5,17]. Significant decreases in fish abundance and diversity with changes in community structure were also observed in these locations [4,5,17]. Furthermore, physiological stress during the 2005 HAB could have altered, or caused the cessation of, normal reproductive activities including the production of sounds typically associated with courtship and spawning [6,9–12]. Additionally, affected animals may have emigrated to areas where bloom conditions were less severe or non-existent [12].

Despite being the most consistently used spawning site in Tampa Bay [9,10], Lowerre-Barbieri and colleagues documented the near total cessation of spotted seatrout sounds in Bunces Pass during the second half of the 2005 spawning season (fig. 6 in [10]). Moreover, Walters *et al.* [12] found that during the 2005 HAB, sand seatrout (*Cynoscion arenarius*) spawning aggregations decreased significantly in lower Tampa Bay where bloom conditions were most severe, whereas they increased in the upper Bay where bloom conditions never reached ichthyotoxic levels [12]. Therefore, fish sound production in lower Tampa Bay was probably affected by a combination of mortality, fish movement and modified/suspended spawning activity as a result of the HAB.

The amplitude of fish chorusing in 2006 appears to be intermediate between the severe HAB year (2005) and later years (2011–2012). This suggests that fish populations or behaviour were relatively slow to recover from the severe HAB conditions in 2005. Previous studies have found that finfish community structure may take a year or more to recover after a severe HAB [4,12,17,18]. For example, Dupont *et al.* [17] found that species richness in areas just offshore of Tampa Bay did not reach original pre-HAB levels until the summer of 2007. While the return of fish sound to maximum levels did not occur in 2006, the amplitude of snapping shrimp sound was higher in 2006 than in later years (2011–2012). It therefore appears that snapping shrimp not only recovered quickly from the 2005 HAB, but also may have drawn some benefit from the resulting ecosystem changes.

Our results show that ambient noise levels from fish and snapping shrimp were significantly lower in summer 2005 during the severe HAB event than in the summers of 2006, 2011 and 2012. We suggest that the decreased sound in 2005 was due to the lethal and/or sublethal effects of the HAB. A dramatic increase in snapping shrimp sounds in 2006, accompanied by a more moderate increase in fish sounds, suggests that fish populations or behaviour take longer to recover from a severe HAB event, while snapping shrimp recover more quickly and may draw some benefit from post-HAB conditions.

This is the first study to identify the potential effects of HABs on snapping shrimp, adding new information to the growing discussion of crustacean susceptibility to brevetoxin exposure and demonstrating the utility of passive acoustic techniques for future monitoring of these phenomena. Additional research investigating cycles of sound production fluctuations, as well as ambient noise levels during HAB events—considering density estimates and behavioural responses of fish and snapping shrimp in relation to concurrent high-resolution *K. brevis* cell counts—would add greatly to our understanding of the ecological dynamics of these events.

## Supplementary Material

RMS Output- the raw RMS values that were used for analyses.

## Data accessibility

Raw RMS values for each 4000 Hz band are accessible in the electronic supplementary material.
